# Combination of nucleated red blood cells and inflammatory biomarkers (PCT and CRP) for predicting sepsis and septic shock in children

**DOI:** 10.3389/fcimb.2025.1603216

**Published:** 2025-08-14

**Authors:** Kaibin Pu, Chunyi Wang, Jie Cheng, Dapeng Chen, Liping Tan, Jihong Tan, Hongdong Li

**Affiliations:** ^1^ Department of Emergency, Children’s Hospital of Chongqing Medical University, Chongqing, China; ^2^ National Clinical Research Center for Child Health and Disorders, Ministry of Education Key Laboratory of Child Development and Disorders, Chongqing, China; ^3^ Chongqing Key Laboratory of Child Rare Diseases in Infection and Immunity, Chongqing, China; ^4^ Department of Neonatology, Children’s Hospital of Chongqing Medical University, Chongqing, China; ^5^ Department of Clinical Laboratory, Children’s Hospital of Chongqing Medical University, Chongqing, China

**Keywords:** C-reactive protein, procalcitonin, nucleated red blood cell, sepsis, septic shock

## Abstract

**Introduction:**

Sepsis remains a leading cause of mortality and morbidity worldwide. This study aimed to investigate the clinical characteristics of children with sepsis and septic shock, with emphasis to evaluate the predictive value of C-reactive protein (CRP), procalcitonin (PCT), and nucleated red blood cell (NRBC) count in pediatric sepsis and septic shock patients.

**Methods:**

We included a total of 121 children, including 80 with sepsis and 41 with septic shock, who were admitted to the Children’s Hospital of Chongqing Medical University between January 2021 and June 2024.

**Results:**

No significant differences in sex, age, weight, or basic diseases were observed between the sepsis and septic shock groups (*P* > 0.05). However, the laboratory findings showed significantly lower platelet counts and hemoglobin levels as well as higher CRP, PCT, NRBC, lactic acid, ALT, CK-MB, urea nitrogen, and APTT levels in the septic shock group (*P* < 0.05). Poorer outcomes were observed in the septic shock group, with higher rates of disease progression or death (63.4% vs. 31.2%, *P* < 0.05). ROC analysis showed that the combination of these three biomarkers achieved greater predictive accuracy (AUC = 0.956), outperforming CRP and PCT alone. Compared with those with sepsis, children with septic shock presented worse clinical and laboratory profiles, required more intensive treatments, and had poorer outcomes.

**Conclussion:**

The inclusion of the NRBC count, combined with CRP and PCT, significantly increases the predictive efficacy of disease severity or progression, particularly indicative of septic shock, highlighting the potential of this combination for early diagnosis and management in pediatric patients.

## Introduction

Sepsis represents a series of clinical syndromes that are caused by a dysregulated host response to infection, which leads to widespread inflammation, tissue damage, and potentially life-threatening multiple organ dysfunction (Singer et al., 2016; Schlapbach et al., 2024). Sepsis poses a significant global health challenge, with approximately 50 million cases reported annually, half of which occur in children under 19 years of age (Rudd et al., 2020). In 2017, the World Health Assembly formally recognized sepsis as a critical focus area for the next decade through a global resolution (Kissoon et al., 2017; Reinhart et al., 2017). Notably, a substantial number of sepsis-related deaths occur shortly after hospital admission, primarily due to severe septic shock and rapid organ failure (Härtel et al., 2020). Furthermore, children who survive sepsis often experience long-term complications, including organ dysfunction or neurodevelopmental impairments (Watson et al., 2024). Therefore, the rapid and accurate recognition of septic shock, followed by timely and effective interventions, is important.

Conventional blood cultures have long been considered the gold standard for diagnosing bacterial sepsis. However, their long processing times and susceptibility to contamination limit their clinical utility. As a result, researchers have used clinical symptoms and laboratory biomarkers to predict or diagnose sepsis and septic shock in pediatric patients. However, the clinical presentation of pediatric sepsis is often ambiguous, and current available laboratory markers lack sufficient sensitivity and specificity, presenting a significant challenge for timely diagnosis (Chiesa et al., 2004).

Current protocols for diagnosing pediatric sepsis and septic shock rely on clinical criteria and biomarkers, such as C-reactive protein (CRP), procalcitonin (PCT), and white blood cell (WBC) counts. While these markers have demonstrated utility in identifying inflammation and infection, their diagnostic accuracy remains limited, especially in pediatric patients. CRP, for instance, is a widely used acute-phase biomarker; however, its delayed elevation—requiring 24–48 hours to peak—reduces its sensitivity in detecting early infections.

Furthermore, CRP is non-specific and may be elevated in a variety of inflammatory conditions, limiting its diagnostic precision. Similarly, PCT, which is an immunologically active protein that is released during systemic inflammatory responses, plays a role in immune regulation and vascular contraction. Its levels increase within 3–4 hours of bacterial infection and peak at 24 hours, making it a more dynamic marker than CRP (Reinhart et al., 2000). However, PCT levels can also increase in noninfectious inflammatory conditions, such as severe trauma or burns, which reduces its specificity. In addition, the high cost and limited availability of PCT testing in primary hospitals and resource-limited settings restrict its routine application.

Recently, nucleated red blood cells (NRBCs) have emerged as a potential biomarker for pediatric sepsis. NRBCs are immature precursors of red blood cells that are not present in the circulation of healthy children (older than 28 days) or adults (healthy newborns have circulating NRBCs that rapidly disappear within a few weeks after birth) (Pedersen et al., 2022). NRBCs help diagnose hematological disorders related to erythropoiesis, anemia, or hemolysis. Recent studies have shown that this economical and easily accessible analysis has potential for the diagnosis and prognosis of critically ill adults and preterm infants (Christensen et al., 2011; Desai et al., 2012; Narcı et al., 2021). However, the value of NRBCs in predicting septic shock remains largely unknown.

Given the limitations of individual conventional biomarkers, combining multiple parameters may enhance diagnostic accuracy and prognostic evaluation. Therefore, this study aimed to analyze the predictive performance of individual biomarkers, including CRP, PCT, and NRBCs, in identifying septic shock. Furthermore, we aimed to compare the predictive performance of NRBCs with that of traditional biomarkers and evaluate the combined predictive utility of CRP, PCT, and NRBCs. By exploring the complementary roles of these biomarkers, we hope to provide a more reliable approach for the early diagnosis and management of pediatric sepsis.

## Materials and methods

### Study design and patients

This retrospective study was conducted at the Children’s Hospital of Chongqing Medical University (Chongqing, China). Pediatric patients who were diagnosed with sepsis at admission between January 2021 and June 2024 were included.

Patients were excluded if they met any of the following criteria: (1) age <28 days or adjusted gestational age <44 weeks; (2) diagnosed with blood disorders (e.g., leukemia, lymphoma, or anemia); (3) congenital or acquired immune deficiencies; or (4) hospitalization duration <72 hours with insufficient clinical documentation. Sepsis and septic shock were defined based on the criteria established by the International Consensus Conference on Pediatric Sepsis (Goldstein et al., 2005) as follows. A diagnosis of sepsis required (1) the presence of ≥2 systemic inflammatory response syndrome (SIRS) criteria; and (2) confirmed or suspected invasive infection. A diagnosis of septic shock required (1) the presence of age-specific SIRS criteria (≥2 items); (2) confirmed or suspected invasive infection; and (3) objective evidence of cardiovascular dysfunction. A diagnosis of cardiovascular dysfunction required documentation of (1) persistent hypotension (age-specific blood pressure values exceeding 2 standard deviations below normative means) following the administration of 40–60 mL/kg fluid resuscitation; (2) the requirement for vasopressor/inotropic support; and (3) objective evidence of hypoperfusion. The threshold of CRP and PCT is 8 mg/L and 0.05 ng/mL, respectively.

This study was approved by the Institutional Review Board of the Children’s Hospital of Chongqing Medical University (No. 133). This study was conducted in accordance with the ethical principles outlined in the Declaration of Helsinki.

### Data collection

Clinical data were extracted from electronic medical records and the hospital’s centralized data platform. The collected variables included demographic characteristics, vasoactive medication use, length of hospitalization, and clinical outcomes. The laboratory parameters at admission included the following: (1) hematological indices: white blood cell count, platelet count, hemoglobin, NRBC; After blood was taken from patients, the number of NRBC was manually counted per high-power microscopic field under a microscope (×400). (2) biochemical markers: troponin, albumin, alanine aminotransferase (ALT), aspartate aminotransferase (AST), blood urea nitrogen (BUN), serum creatinine (Scr), potassium, sodium, calcium, and glucose, and lactate; (3) inflammatory markers: CRP and PCT; (4) coagulation profiles: international normalized ratio (INR) and activated partial thromboplastin time (APTT); and (5) microbiological data: pathogen culture results. The outcomes were stratified into two categories: (1) favorable: sepsis resolution with hospital discharge; (2) unfavorable: disease progression despite treatment, severe sequelae, treatment withdrawal, and sepsis-related mortality.

### Statistical analysis

The data distribution for continuous measures are summarized using median values with interquartile ranges (IQRs). Comparative assessments of proportions across categorical parameters were conducted via χ2 or Fisher’s exact tests based on sample size adequacy. Between-group differences in non-normally distributed variables were evaluated through Wilcoxon rank-sum tests, whereas parametric comparisons utilized independent samples t tests. ROC curve analyses were performed for CRP, PCT, and NRBC individually and in combination, with optimal cutoffs derived by maximizing Youden’s index. All the computational workflows and visualizations were executed in SPSS 26.0 (IBM Corp., Armonk, NY, USA). P values less than 0.05 were predefined as statistically significant thresholds.

## Results

### Demographic characteristics

The present study enrolled 121 children, including 80 patients with sepsis and 41 patients with septic shock. The demographic characteristics of the study participants are presented in **Table 1**. No significant differences were observed between the sepsis and septic shock groups in terms of sex, age, weight, disease course or basic disease. A significantly greater proportion of patients in the septic shock group had a history of surgery compared to the sepsis group (73% vs. 35%, *P* < 0.05). The sources of infection varied between the two groups, with respiratory tract infections being more prevalent in the septic shock group than in the sepsis group (65.8% vs. 49%, *P* < 0.05), whereas bloodstream infections were less common (2.4% vs. 12.5%, *P* < 0.05).

**Table 1:** Demographic characteristics in children with bacterial sepsis.

	Sepsis (N=80)	Septic shock (N=41)	*P*-value
Age, month Sex, n (%)	35 (2.7-192)	41 (2.2-185)	0.32
Female	47 (59)	24 (59)	0.26
Male	33 (41)	17 (41)	0.19
Weight, kilograms	15 (6-41)	17 (5.2-37.8)	0.34
Course of disease, days	4.2 (1-10)	4.8 (2-7.3)	0.43
Basic disease, n (%)	47 (59)	28 (68)	0.19
Surgical History, n (%)	28 (35)	30 (73)	0.02
Source of infection, n (%)			
Abdomen	15 (19)	5 (12.2)	<0.001
Respiratory tract	39 (49)	27 (65.8)	0.04
Blood stream	10 (12.5)	1 (2.4)	0.02
Urinary tract	3 (3.5)	4 (9.8)	0.01
Soft tissue	2 (2.5)	0 (0)	0.02
Others	11 (13.5)	4 (9.8)	0.07
Positive blood culture	10 (12.5)	15 (36.5)	<0.001

NRBC, Nucleated red blood cell.

Categorical variables are expressed as absolute numbers (percentage). Continuous variables are expressed as median (interquartile range).

### Laboratory indicators

**Figure 1:**
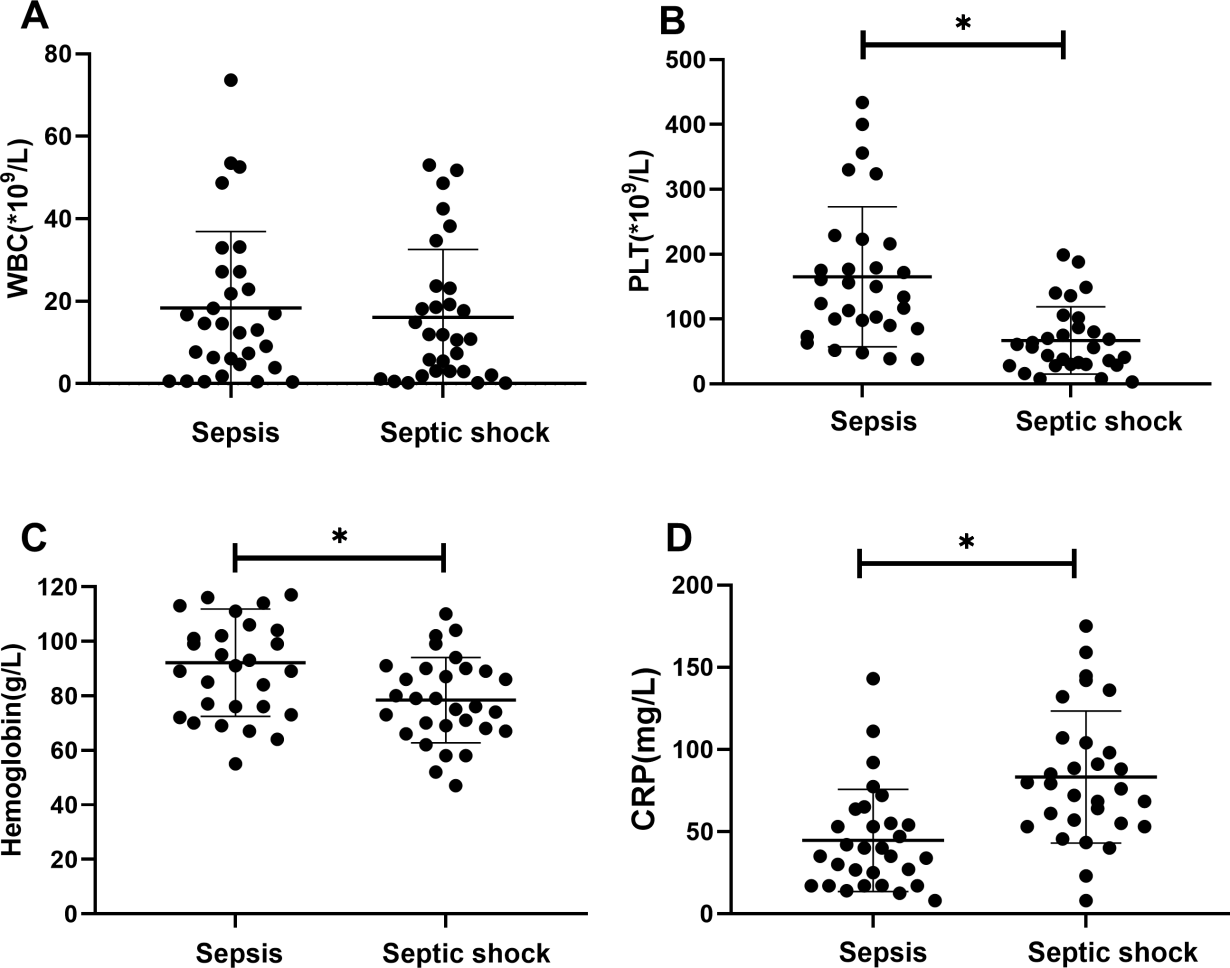
Blood index for septic children with and without nucleated red blood cells (NRBCs) before and after treatment. WBC **(A)**, PLT **(B)**, Hemoglobin **(C)** and CRP **(D)** levels present in the blood of the respective groups are shown. Asterisk denotes a statistically significant difference between sepsis group and septic shock group (*P* < 0.05). WBC, white blood cell; PLT, platelet; CRP, c-reactive protein.

**Figure 2:**
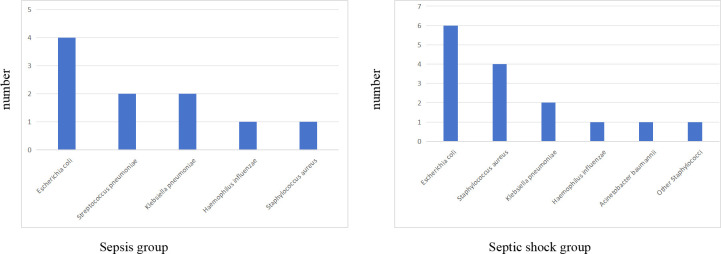
The bacterial species distribution of blood cultures in sepsis group and septic shock group.

**Figure 3:**
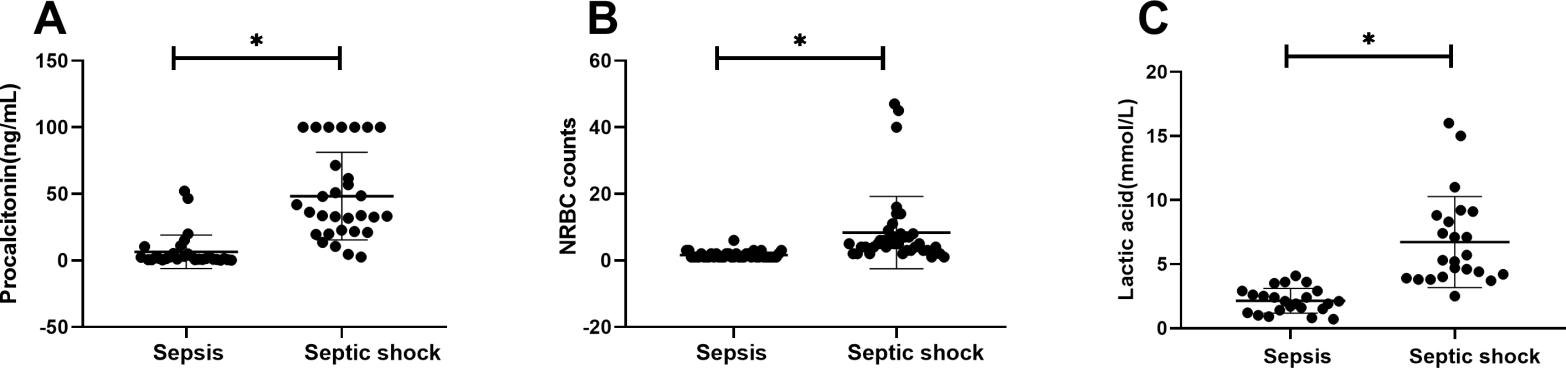
Laboratory values for children of sepsis group and septic shock group. Procalcitonin **(A)**, NRBC counts **(B)** and lactic acid **(C)** levels present in the blood of the respective groups are shown. Asterisk denotes a statistically significant difference between sepsis group and septic shock group (*P* < 0.05). NRBC, nucleated red blood cell.

**Figure 4:**
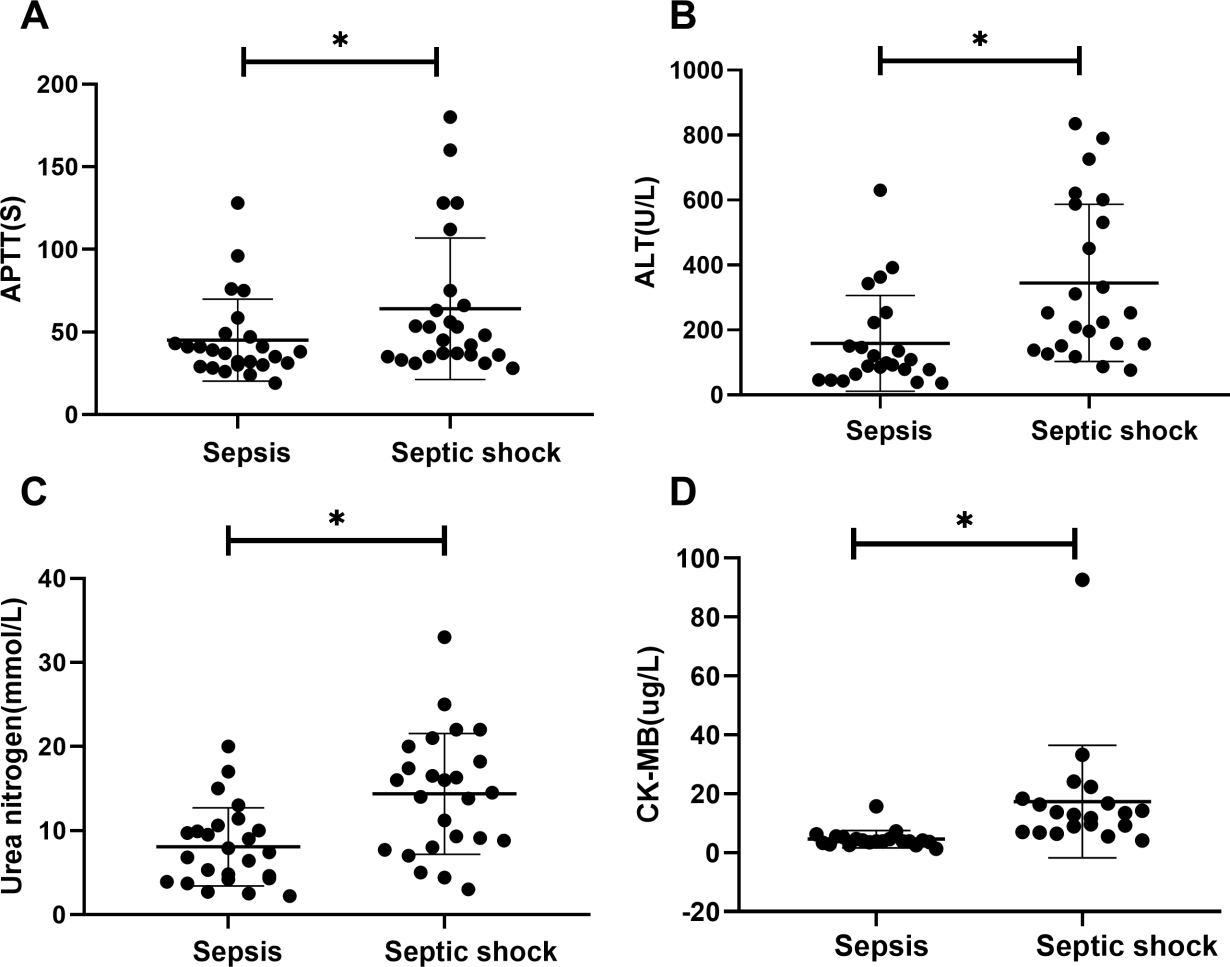
Laboratory values for children of sepsis group and septic shock group. APTT **(A)**, ALT **(B)**, urea nitrogen **(C)** and CK-MB **(D)** levels present in the blood of the respective groups are shown. Asterisk denotes a statistically significant difference between sepsis group and septic shock group (*P* < 0.05). APTT, activated partial thromboplastin time; ALT, alanine aminotransferase; CK-MB, creatine kinase muscle and brain isoenzyme.

There were statistically significant differences in laboratory indicators between the sepsis and septic shock groups. In terms of blood parameters, platelet count and hemoglobin levels were significantly lower, whereas the CRP levels were significantly higher in the septic shock group than in the sepsis group (*P* < 0.05) (**Figure 1**). WBC counts were not significantly different between the two groups. In addition, positive blood culture rates were significantly higher in the septic shock group than in the sepsis group (36.5% vs. 12.5%) (*P* < 0.001) (**Table 1**). The most common etiology of blood cultures was *Escherichia coli* in both groups (**Figure 2**). For biochemical markers, PCT, NRBC, lactic acid, ALT, CK-MB, urea nitrogen, and APTT levels were significantly higher in the septic shock group than in the sepsis group (*P* < 0.05) (**Figures 3**, **4**).

### Treatment and clinical outcomes in children with sepsis

The treatment of sepsis and septic shock followed the guidelines established by the International Consensus Conference on Pediatric Sepsis ^[14]^. The proportion of patients requiring invasive mechanical ventilation was significantly higher in the septic shock group than in the sepsis group (100% vs. 27.5%, *P* < 0.001). Conversely, the proportion of patients requiring non-invasive or no respiratory support was significantly lower in the septic shock group than in the sepsis group (0% vs. 10%, P = 0.02; 0% vs. 62.5%, *P* < 0.001). Patients in the septic shock group were more likely to undergo blood transfusions (34/41, 83% vs. 45/80, 56%, *P* < 0.001) and hemopurification (28/41, 68.3% vs. 14/80, 17.5%, *P* < 0.001) (**Table 2**). The septic shock group had longer hospital stays than did the sepsis group (median 20.2 days vs. median 16.4 days, *P* < 0.05). Additionally, a greater proportion of patients in the sepsis group improved after treatment (68.8% vs. 36.6%, *P* < 0.05), whereas 63.4% of children in the septic shock group experienced disease progression or even death.

**Table 2:** Treatment and clinical outcomes in children with sepsis.

	Sepsis (N=80)	Septic shock (N=41)	*P*-value
Surgery during hospitalization, n (%) Respiratory support, n (%)	16 (20)	4 (9.8)	0.25
Invasive	22 (27.5)	41 (100)	<0.001
Non-invasive	8 (10)	0 (0)	0.02
None	50 (62.5)	0 (0)	<0.001
Transfusion, n (%)	45 (56)	34 (83)	<0.001
Use of hemopurification, n (%)	14 (17.5)	28 (68.3)	<0.001
Hospital stay, days	16.4 (10-23.2)	20.2 (15.5-30.8)	0.01
Outcomes, n (%)			
Improvement	55 (68.8)	15 (36.6)	0.03
Poor and death	25 (31.2)	26 (63.4)	0.01

NRBC, Nucleated red blood cell.

Categorical variables are expressed as absolute numbers (percentage). Continuous variables are expressed as median (interquartile range).

### ROC curve analysis

**Figure 5:**
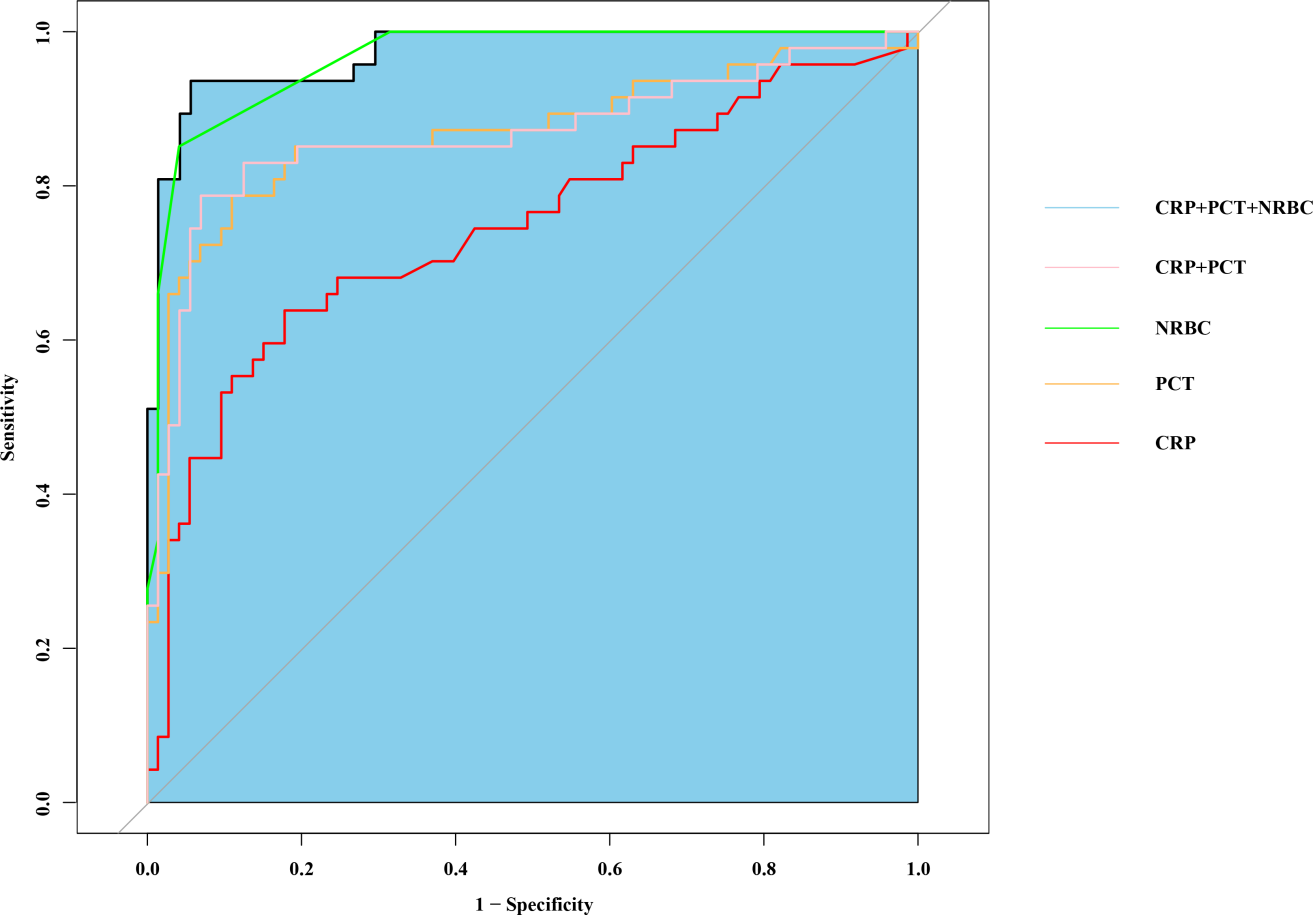
Combination of nucleated red blood cell, PCT and CRP to predict septic shock by receiver operating characteristic (ROC) curves. PCT, procalcitonin; CRP, c-reactive protein.

The diagnostic accuracy of CRP, PCT, and the NRBC for the prediction of septic shock in children was assessed. The areas under the curve (AUCs) of the receiver operating characteristic (ROC) curves of CRP, PCT and the NRBC count for the prediction of sepsis in children were 0.746 [95% confidence interval (CI) 0.650–0.842], 0.868 [95% CI 0.818–0.912] and 0.923 [95% CI 0.857–0.944], respectively. The AUC for the combination of CRP, PCT, and the NRBC count for the prediction of septic shock in children was 0.956 [95% CI 0.873–0.971] (**Figure 5**, **Table 3**). Compared to CRP or PCT alone, and combination of these two biomarkers, the combination of CRP, PCT and NRBC achieved a greater predictive accuracy.

**Table 3:** Analysis of the predictive value of relevant indicators in patients with septic shock.

Indicators	AUC	95% CI	Cut-off	Sensitivity (%)	Specificity (%)
CRP	0.746	0.650-0.842	43.5	78.1	76.7
PCT	0.886	0.818-0.912	7.785	87.6	83.7
NRBC	0.923	0.857-0.944	3	90.7	88.4
CRP+PCT	0.897	0.871-0.948	/	89.1	85.6
CRP+PCT+NRBC	0.956	0.873-0.971	/	93.3	88.5

CRP, c-reactive protein; PCT, procalcitonin; NRBC, Nucleated red blood cell; AUC, areas under the curve.

## Discussion

This study evaluated the individual and combined diagnostic accuracies of CRP, PCT and NRBCs for predicting the occurrence and severity of sepsis in the pediatric population. Our findings revealed that NRBC counts, when analyzed alongside CRP and PCT levels, are valuable biomarkers for distinguishing between sepsis and septic shock. The combination of these three biomarkers demonstrated superior predictive accuracy compared with CRP or PCT alone, highlighting the enhanced diagnostic potential of this panel. These results emphasize the role of NRBCs as a complementary biomarker that not only improves diagnostic precision but also reflects the physiological stress and disease severity associated with sepsis.

Sepsis is a life-threatening condition that imposes a substantial burden worldwide. According to the Global Burden of Disease (GBD) study, approximately 25 million sepsis cases occur annually in pediatric and neonatal populations, resulting in approximately 3 million deaths (Watson et al., 2024). The absence of definitive biomarkers for the timely diagnosis of sepsis exacerbates the inappropriate use of antibiotics. Therefore, identifying a potential combination of biomarkers to improve the early diagnosis of sepsis is imperative.

In this study, the positive rates of blood culture in sepsis and septic shock patients were 12.5% and 36.5%, respectively. The most common bacterial isolate was *Escherichia coli*. These findings differ from those of a meta-analysis by Droz et al (Droz et al., 2019), which reported a pooled blood culture positivity rate of 28% (95% CI: 13.2–45.8%) among pediatric populations in low- and middle-income Asian countries. The lower positivity rate observed in our sepsis cohort may reflect differences in patient demographics, clinical settings, or regional pathogen prevalence. Although blood culture is considered the gold standard for diagnosing sepsis, its sensitivity is influenced by multiple factors. For example, delays in sample collection, previous antibiotic administration, technical limitations of detection methods, and insufficient blood sample volume all contribute to variability in positive rates (Lambregts et al., 2019; Huber et al., 2020; Whelan et al., 2024). These challenges are particularly pronounced in pediatric patients, where obtaining adequate blood volumes for culture is often more difficult than in adults.

Our analysis revealed significantly higher CRP concentrations in patients with septic shock than in those diagnosed with sepsis (*P* < 0.05). However, CRP has shown limited diagnostic reliability because of its gradual elevation during the early phases of infection and its frequent elevation in noninfectious conditions. In this study, the ROC curve analysis revealed that plasma CRP level exhibited the poorest diagnostic performance, with low specificity in the early diagnosis of sepsis in children. Current evidence suggests integrating CRP with other markers to optimize diagnostic accuracy rather than employing it as a standalone tool for sepsis screening (Morad et al., 2020).

Serum PCT is widely recognized as a biomarker of severe bacterial infections and the early diagnosis of sepsis, as well as for monitoring disease progression in infectious conditions. A meta-analysis by Menon et al. on severity-related variables in children demonstrated that PCT levels are positively correlated with mortality rates, which can serve as a predictive indicator for septic shock patients (Menon et al., 2022). In the present study, PCT levels were significantly higher in the septic shock group than in the sepsis group (*P* < 0.05). One study reported that a procalcitonin threshold of 19.1 ng/mL was associated with septic shock in children with sepsis (Rey et al., 2007). In this study, using a cutoff value of PCT ≥7.785 μg/L determined by the ROC curve, PCT demonstrated a sensitivity of 87.6% and specificity of 83.7% (**Table 3**). In pediatric septic shock patients, procalcitonin demonstrated superior predictive capability relative to CRP, a finding consistent with established clinical research (Rey et al., 2007; Wacker et al., 2013; Morad et al., 2020).

Early recognition and differentiation of sepsis and septic shock are critical for initiating timely and appropriate management. However, diagnostic challenges persist owing to nonspecific clinical presentations and the limitations of existing laboratory tests. These limitations highlight the urgent need for novel biomarkers that can increase diagnostic accuracy, allow for early risk stratification, and guide personalized treatment strategies. Our study addresses this need by demonstrating that NRBCs improve the diagnostic performance of traditional biomarkers.

NRBCs represent immature erythroid precursors that enter circulation during severe physiological stressors such as hypoxia, systemic inflammation, or bone marrow activation (Pedersen et al., 2022). Their presence in circulation beyond the neonatal period is considered pathological and has been associated with poor outcomes in critically ill patients. In this study, NRBC counts were significantly higher in children with septic shock than in those with sepsis (*P* < 0.05). Our findings confirm the prognostic utility of NRBC counts in pediatric septic shock. These results confirm the prognostic value of NRBC quantification for mortality risk stratification in pediatric patients with septic shock, confirming previous the results of previous studies (Pedersen et al., 2022; Noor et al., 2023; Kirsch et al., 2024).

Previous work from our group identified NRBC positivity as an independent risk factor for disease severity and mortality in pediatric sepsis (Li et al., 2023). In the adult population, Desai et al. observed that surgical sepsis patients with detectable NRBCs exhibited significantly elevated mortality risks in both ICU and general ward settings. Their threshold analysis revealed mortality rates exceeding 50% at NRBC peaks >500/μL, with survival rates dropping below 12% for counts >2000/μL (Desai et al., 2012). Narcı et al.’s retrospective analysis also established NRBC counts as independent predictors of mortality across trauma, sepsis, and critically ill populations (Narcı et al., 2021). Collectively, these findings substantiate NRBC elevation as a biomarker of pathophysiological dysregulation proportional to disease severity.

In this study, when a cutoff value of NRBC count ≥ 3 was used, as determined by ROC curve analysis, the NRBC count demonstrated a sensitivity of 90.7% and a specificity of 88.4% (**Table 3**). When combined with CRP and PCT, the NRBC count improved the risk prediction accuracy (AUC increased from 0.746 to 0.956), enabling more precise clinical assessment in pediatric sepsis management. This combined biomarker strategy facilitates earlier recognition of sepsis and severity stratification, thereby supporting time-sensitive clinical decisions regarding therapeutic escalation.

The pathophysiological mechanisms underlying elevated NRBC counts in pediatric sepsis and septic shock patients require further investigation. Under conditions of hypoxia, systemic inflammation, or tissue damage, signaling molecules such as erythropoietin and interleukin proteins (e.g., interleukin-3 and interleukin-6 [IL-6]) are activated (Stachon et al., 2005; Kirsch et al., 2024). Experimental evidence has identified IL-6 as a key regulator that promotes erythropoietin synthesis during systemic inflammatory response syndrome following septic shock in pediatric populations (Krafte-Jacobs and Bock, 1996). These biochemical mediators stimulate the maturation of immature red blood cells in the bone marrow, leading to the appearance of NRBCs in the circulation. Our findings suggest that this phenomenon is likely related to tissue hypoxia caused by systemic hypoperfusion.

In our study, children with septic shock required more mechanical ventilation and had higher lactate levels than those with sepsis alone did, supporting the role of oxygen deprivation in NRBC production. Additionally, elevated CRP and PCT levels in children with septic shock further indicate the involvement of inflammatory mediators in the synthesis and release of NRBCs. Further research is needed to elucidate the mechanisms underlying this association.

This study has several limitations. First, the study population was drawn from a single center, resulting in a relatively small sample size, which may limit the generalizability of our findings. In addition, the retrospective design of the study may have introduced bias in data recording and collection. Further research with larger sample sizes and multicenter designs is needed to validate these findings. At last, the future prospective studies are required to validate the mechanism of NRBC sources, due to the effects of anemia or hypoxia.

## Conclusions

In conclusion, this study demonstrated that the NRBC count combined with the CRP and PCT levels have value for the diagnosis and prognosis of pediatric sepsis and septic shock. This biomarker combination improves the ability to reliably distinguish between sepsis and the progression of septic shock, representing a substantial advancement in sepsis care. By enabling earlier detection, more precise risk stratification, and more effective therapeutic interventions, the findings of the present study help address critical challenges in the management of pediatric sepsis. Given the global burden of pediatric sepsis and the limitations of existing diagnostic tools, incorporating NRBC measurements into clinical practice has the potential to transform sepsis management and improve outcomes for children worldwide. Future research should aim to validate these findings in larger, multicenter studies, explore their applicability across diverse populations, and investigate the broader implications of NRBC measurement for sepsis management and public health strategies.

## Data Availability

The raw data supporting the conclusions of this article will be made available by the authors, without undue reservation.
